# Network analysis and *in vivo* experiments reveal the therapeutic mechanisms of total ginsenosides in a *Drosophila* model of ulcerative colitis

**DOI:** 10.3389/fphar.2025.1556579

**Published:** 2025-03-25

**Authors:** Gongchen He, Jian Sun, Yuexin Gu, Yanjie Zheng, Liang Wang, Yanyan Sun

**Affiliations:** ^1^ College of Food Science and Engineering, Northwest A&F University, Yangling, Shaanxi, China; ^2^ Institute of Agricultural Quality Standard and Testing Technology, Jilin Academy of Agricultural Sciences, Changchun, Jilin, China; ^3^ Soybean Research Institute, Jilin Academy of Agricultural Sciences, Changchun, Jilin, China

**Keywords:** total ginsenosides, ulcerative colitis, network analysis, *Drosophila melanogaster*, intestinal homeostasis

## Abstract

Gut homeostasis is critical for human health, ulcerative colitis (UC) can disrupt gut homeostasis and cause disease. *Panax ginseng* C.A. Meyer is a widely used traditional herbal medicine known for its anti-inflammatory, antioxidant, and immunomodulatory effects. However, the protective mechanisms of total ginsenosides (TG) in treating UC remain unclear. In this study, we employed *Drosophila* melanogaster as a model organism to investigate the protective effects of TG on dextran sulfate sodium (DSS)-induced intestinal injury. Our data showed that TG significantly improved survival rates in female flies, restored intestinal length, maintained intestinal barrier integrity, and alleviated oxidative stress. Additionally, TG may protect against intestinal damage by activating the PI3K/Akt signaling pathway and inhibiting the JAK/STAT signaling pathway. These findings suggest that TG alleviates UC symptoms through multi-target regulation, highlighting its potential for developing novel therapeutic strategies for UC.

## Graphical Abstract

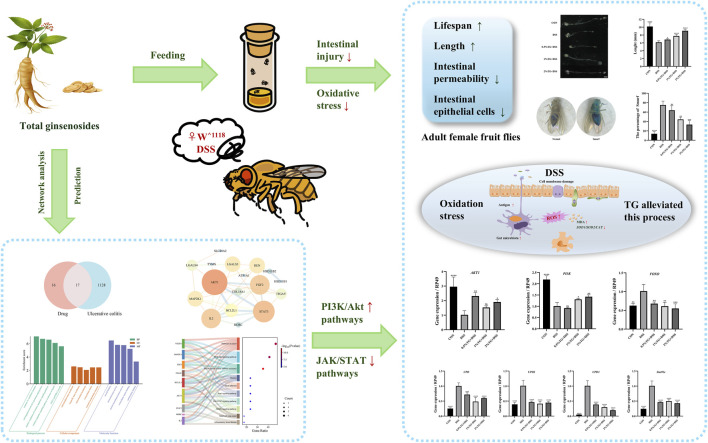

## 1 Introduction

Recently, the incidence of inflammatory bowel disease (IBD) has shown a marked increase, particularly among younger individuals (Hendler et al., 2020). Notably, the pathophysiology of IBD is intricate and multifaceted and involves the interplay of various factors, including genetic predispositions, environmental influences, epithelial barrier dysfunction, disruption of the gut microbiota, immune system imbalances, and oxidative stress damage (Guan, 2019). Ulcerative colitis (UC), a primary subtype of IBD, is clinically characterized by symptoms such as diarrhea, abdominal pain, and the presence of mucus or blood in the stools (Shen et al., 2019). Currently available UC therapies primarily include immunosuppressants, anti-inflammatory agents, and biologics (Gros and Kaplan, 2023). Although these medications are effective, they are frequently accompanied by adverse effects such as immune suppression and gut microbiota dysbiosis. Additionally, prolonged use may result in drug resistance or undesirable side effects, including gastrointestinal discomfort and osteoporosis (Akkol et al., 2020). Consequently, identifying natural plant-derived bioactive metabolites with high efficacy, low toxicity, and minimal side effects for the treatment of IBD has attracted research attention.

In China, botanical drug therapies have long played a prominent role in the treatment of UC, earning widespread recognition for their low toxicity, minimal side effects, and affordability (Xue et al., 2023). For over 2000 years, *Panax ginseng* C.A. Meyer, a plant belonging to the *Panax* genus of the *Araliaceae* family, has been highly regarded not only as a tonic but also for its diverse therapeutic properties, which have been scientifically substantiated in the treatment of various conditions, including inflammatory (Zhao et al., 2024), neurological (Zhao et al., 2022), and oncological (Xia et al., 2024) diseases. It is noteworthy that traditional Chinese medicine formulations, predominantly centered on ginseng—such as Shenling BaiZhu powder and Six Gentlemen Decoction—are frequently employed in the clinical management of ulcerative colitis and have been shown to yield significant therapeutic benefits (Chen et al., 2022; Yuan et al., 2020). Ginseng contains various bioactive compounds, including ginsenosides, polysaccharides, and peptides, with ginsenosides being the most abundant (Kim et al., 2017). Accumulating evidence suggests that ginsenosides exert physiological effects in the treatment of inflammatory diseases through multiple processes, such as suppressing the release of pro-inflammatory mediators (Zhao et al., 2024), modulating gut microbiota (Liu et al., 2024), and enhancing immune function (Zhu et al., 2021). Collectively, these actions underscore the clinical potential of ginsenosides for mitigating inflammation and modulating immune responses. Additionally, ginsenosides have been used to treat cardiovascular diseases, diabetes, and various cancers (Wei et al., 2024). Despite the promising pharmacological effects of ginsenosides, their anti-inflammatory and antioxidant effects and their mechanism of action in UC remain unclear. A comprehensive investigation of the potential therapeutic effects and mechanisms of action of ginsenosides in UC may provide novel insights and avenues for treatment.

Agents commonly used to induce colitis include dextran sulfate sodium (DSS), trinitrobenzene sulfonic acid, and sodium dodecyl sulfate. Among these, DSS is widely used because of its ability to rapidly induce intestinal inflammation with pathological features closely resembling those of human UC, making it a valuable tool for exploring the mechanisms of colitis and evaluating the efficacy of potential therapeutics (Yang and Merlin, 2024). Recently, the fruit fly (*Drosophila melanogaster*) has emerged as an alternative model for colitis research because of its simple genome, short reproductive cycle, low cost, and anatomical and signaling pathway similarities to humans and other mammals (Casali and Batlle, 2009; Medina et al., 2022). For example, San Huang Pill and its bioactive components alleviated DSS-induced colitis in *Drosophila* by regulating pathways such as JAK/STAT, apoptosis, Toll, and Nrf2/Keap1 (Li et al., 2024). Similarly, bilberry anthocyanin extracts exerted protective effects against DSS-induced intestinal damage in *Drosophila* by reducing reactive oxygen species (ROS) and malondialdehyde (MDA) levels and activating the NRF2 signaling pathway (Zhang et al., 2022). Overall, these findings highlight the unique advantages of *Drosophila* in revealing the pathophysiological mechanisms of intestinal inflammation. Additionally, the high manipulability and experimental reproducibility of this model make it an important tool for investigating the mechanisms of action of botanical drug in gut inflammation.

Network pharmacology allows for the analysis of the biological effects of drug components, the prediction of involved signaling pathways, the identification of regulatory genes, and the revelation of multi-target and multi-pathway action patterns (Kim et al., 2024). This approach not only facilitates a deeper understanding of the multidimensional regulatory interactions between drugs and diseases but also provides valuable insights for exploring their mechanisms of action.

Therefore, we investigated the protective effect and regulatory mechanisms of total ginsenosides (TG) using a *Drosophila* model of DSS-induced intestinal injury and network analysis. Overall, this study will improve our understanding of the potential anti-inflammatory and antioxidative roles of TG in UC, thereby providing a theoretical basis for its clinical application in UC management.

## 2 Materials and methods

### 2.1 Experimental drugs

TG extracted from root of *Panax ginseng* C. A. Mey. (purity: 80.22%, S25997, batch number: H12 M8T35871) and 4,6-diamidino-2-phenylindole (DAPI) were purchased from Yuanye Bio-Technology Co., Ltd., Shanghai, China. DSS salt was purchased from MP Biomedicals Co., Ltd., United States; dihydroethidium (DHE) was purchased from Bairuiji Biotechnology Co., Ltd., Beijing, China; 7-aminoactinomycin D (7-AAD) was purchased from Beyotime Biotechnology Co., Ltd., Shanghai, China; and MDA assay kit was supplied by Solarbio Bioscience and Technology Co., Ltd., Shanghai, China.

### 2.2 *Drosophila* strains and rearing

The *w* ^*^1118*^ *Drosophila* strain used in this study was obtained from the Tsinghua Fly Center. The flies were reared on standard cornmeal-yeast medium in a controlled environment (temperature, 25°C; relative humidity, 60%) under a 12 h/12 h light/dark cycle.

### 2.3 Establishment of *Drosophila* model of UC

The *Drosophila* model of UC was established using DSS. Briefly, adult female flies aged 3–7 days were anesthetized with CO_2_ and randomly divided into five groups (20 flies per group): control, 5% DSS, 0.5% TG + 5% DSS, 1% TG + 5% DSS, and 2% TG + 5% DSS. Each of the prepared solutions (60 μL) was uniformly applied to the surface of the standard medium and replaced every 24 h.

### 2.4 Survival assay

Briefly, the collected flies were separated by sex into female and male groups (three vials per group) and transferred to the respective treatment media. The mortality in each vial was recorded every 24 h.

### 2.5 Intestinal morphology assays

During the experimental period, female flies in the control group were administered sterile water, whereas those in the other groups were treated for 72 h with either 5% DSS alone or 5% DSS combined with 0.5, 1, or 2% TG. After treatment, the flies were starved for 1 h and the entire intestine was dissected and extracted in ice-cold PBS. Thereafter, the intestines were observed and imaged using a stereofluorescence microscope (Nikon). Intestinal lengths were measured using the ImageJ software (version 1.8.0, ×64, NIH, Bethesda, Rockville, MD, United States).

### 2.6 “Smurfs” assay

Intestinal integrity was assessed using the Smurf assay. Briefly, bright blue dye (FD and C Blue #1) was added to the standard medium at a concentration of 2% (w/v). Each group of flies was first raised on media with or without 5% DSS for 5 days and transferred to blue dye medium for 12 h of incubation. A fly was identified as a “Smurf” if blue dye was observed outside of its digestive tract. Finally, the leakiness rate was calculated based on the percentage of blue-colored flies in the total population.

### 2.7 Determination of intestinal epithelial cell death in flies

After 72 h of induction, the intestines of female flies were dissected in cold phosphate-buffered saline (PBS) and stained with 7-AAD at room temperature (25°C) for 30 min. After washing with PBS for 5 min, the intestines were stained with DAPI for 20 min and washed again in PBS for 5 min. Finally, the tissues were mounted with an anti-fade reagent and observed using a motorized inverted fluorescence microscope (Olympus, Tokyo, Japan). Images were processed using ImageJ software (NIH).

### 2.8 Reactive oxygen species assay

After maintaining the flies in media with or without 5% DSS for 72 h, the intestines were dissected in cold PBS and incubated with 20 μM DHE for 10 min. Thereafter, the tissues were washed with PBS for 5 min, stained with DAPI for 20 min, washed with PBS for another 5 min. Slides were mounted using an anti-fade reagent and observed using a confocal microscope (Leica, Germany).

### 2.9 Antioxidant capacity assays

Briefly, 120 flies were collected from each treatment group after 72 h, fasted for 2 h, and frozen at ˗20°C for 1 h before weighing. Thereafter, the flies were homogenized in an ice bath with saline solution at a 1:9 ratio (fly weight: saline volume) and centrifuged at 8,000 *g* for 10 min at 4°C. MDA content was measured using a commercial assay kit (Solarbio, Shanghai, China; Cat# BC0025) following the manufacturer’s instructions.

### 2.10 Target identification of major TG metabolites

Based on a previous report (Su et al., 2023), the primary ginseng saponins in TG are Rg1, Re, Rb1, Rc, Rb2, Rd, and Rf. To obtain relevant information on these active components, data were retrieved from the PubChem database (https://pubchem.ncbi.nlm.nih.gov/) (Table 1). Thereafter, the obtained chemical structures were submitted to the SwissTargetPrediction database (http://www.swisstargetprediction.ch/) to predict the potential targets.

**TABLE 1:** Major ginseng saponins in ginseng saponins.

No.	Compound	Cas no.	Molecular formula	Molecular structure
Rb1	Ginsenoside Rb1	41,753-43-9	C_54_H_92_O_23_	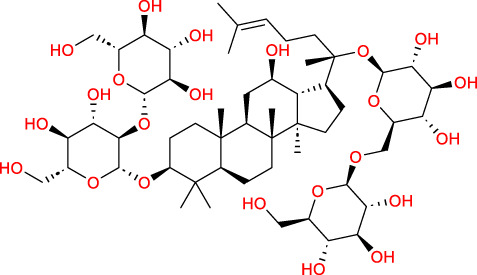
Rb2	Ginsenoside Rb2	11,021-13-9	C_53_H_90_O_22_	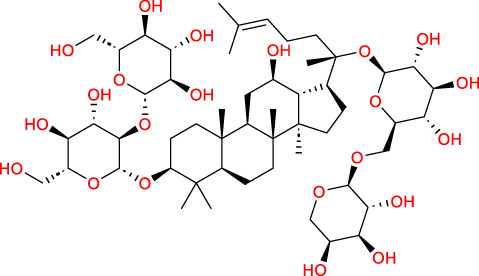
Rc	Ginsenoside Rc	11,021-14-0	C_53_H_90_O_22_	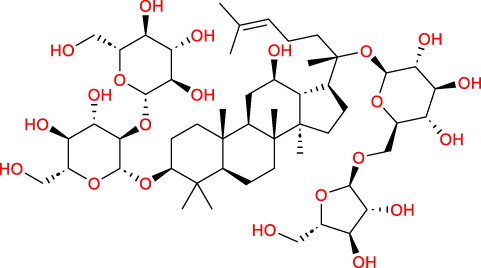
Rd	Ginsenoside Rd	52,705-93-8	C_48_H_82_O_18_	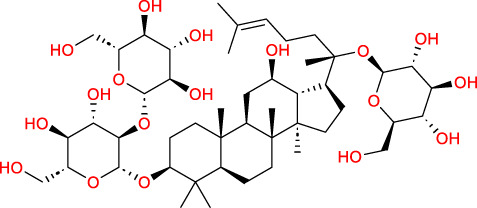
Re	Ginsenoside Re	52,286-59-6	C_48_H_82_O_18_	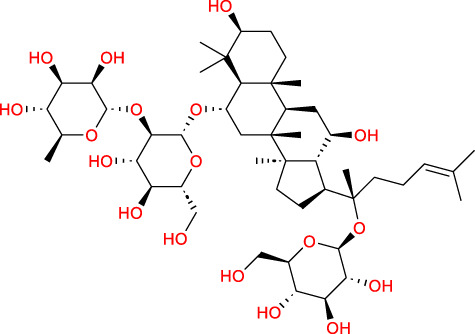
Rf	Ginsenoside Rf	52,286-58-5	C_42_H_72_O_14_	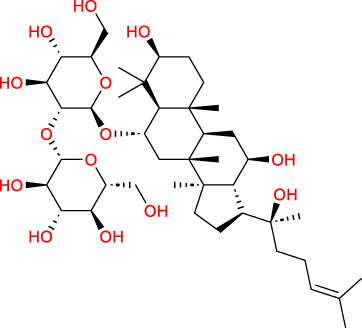
Rg1	Ginsenoside Rg1	22,427-39-0	C_42_H_72_O_14_	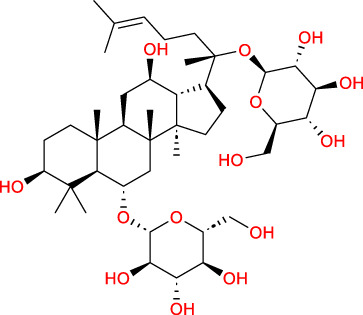

### 2.11 UC related targets and hub target

To identify UC-related targets, we searched the term “ulcerative colitis” in multiple databases: GeneCards (https://www.genecards.org/), OMIM (https://www.omim.org/), TTD (https://db.idrblab.net/ttd/), DrugBank (https://go.drugbank.com/), and PharmGKB (https://www.pharmgkb.org/). Thereafter, the obtained targets were merged, deduplicated, and summarized to create a comprehensive UC target database. The UniProt protein database (https://www.uniprot.org) was used to standardize both disease-related and potential TG component targets for Gene Symbols. Finally, these genes were mapped to identify potential TG targets for UC.

### 2.12 Protein-protein interaction (PPI) analysis

A PPI network was used to explore the associations between compound- and disease-related proteins. To elucidate the role of target proteins at the system level, the identified targets were uploaded to the STRING database (https://string-db.org), with “*Homo sapiens*” as the selected species and a confidence level threshold set at 0.70 to ensure robust target associations. Thereafter, the PPI network data were visualized and analyzed using the CytoScape software (version 3.7.2, × 64, Cytoscape Consortium, San Diego, CA, United States) to generate a comprehensive network map.

### 2.13 Gene ontology (GO) and kyoto encyclopedia of genes and genomes (KEGG) analyses

To elucidate functions and pathways of the target genes of TG, we performed GO functional annotation and KEGG pathway enrichment analysis using the Metascape platform (http://metascape.org/gp/index.html). The results were visualized and graphically represented using the bioinformatics platform (http://www.bioinformatics.com.cn/).

### 2.14 Real-time quantitative polymerase chain reaction (RT-qPCR)

After the various treatments for 72 h, total RNA was extracted from the intestines of 30 female flies using TRIzol reagent and quantified using a microvolume spectrophotometer. Thereafter, the RNA was reverse-transcribed to generate cDNA using an Evo M-MLV RT premix kit. RT-qPCR was performed on the QuantStudio 6 Flex system using specific reagents and primers. Gene expression changes were analyzed using the $2^{-∆∆CT}$ method (Livak and Schmittgen, 2001), with *RP49* as the reference gene. The primer sequences are listed in Table 2.

**TABLE 2:** RT–qPCR primers.

Gene name	Forward primer 5′-3′	Reverse primer 5′-3′
*RP49*	CTTCATCCGCCACCAGTC	GCA​CCA​GGA​ACT​TCT​TGA​ATC
*SOD1*	GCGGCGTTATTGGCATTG	ACT​AAC​AGA​CCA​CAG​GCT​ATG
*SOD2*	CAC​ATC​AAC​CAC​ACC​ATC​TTC	GCTCTTCCACTGCGACTC
*CAT*	TGA​ACT​TCC​TGG​ATG​AGA​TGT​C	TCT​TGG​CGG​CAC​AAT​ACT​G
*PI3K*	CCTGCTTGGCGACTATCC	CGT​TGT​GGT​GGT​TGA​TGT​AG
*Akt-1*	GCT​ATG​ACG​CCA​TCT​GAA​C	CGCCGCTGCTATTACAAG
*FOXO*	CCT​CAT​CCA​ATG​CCA​GTT​C	TGCGTCATCGTTGTGTTC
*UPD*	CCA​CGT​AAG​TTT​GCA​TGT​TG	CTA​AAC​AGT​AGC​CAG​GAC​TC
*UPD2*	CGG​AAC​ATC​ACG​ATG​AGC​GAA​T	TCG​GCA​GGA​ACT​TGT​ACT​CG
*UPD3*	GAG​CAC​CAA​GAC​TCT​GGA​CA	CCA​GTG​CAA​CTT​GAT​GTT​GC
*Stat92e*	CTG​GGC​ATT​CAC​AAC​AAT​CCA​C	GTA​TTG​CGC​GTA​ACG​AAC​CG

### 2.15 Statistical analysis

Data analysis and figure generation were performed using GraphPad Prism 9.5 (San Diego, CA, United States). Additionally, image analysis was conducted with ImageJ (NIH). Significant differences between groups were determined using Student’s t-test. Statistical significance was set at *P* < 0.05 (significant) or *P* < 0.01 (highly significant). All experiments were conducted at least three times to ensure data reliability.

## 3 Results

### 3.1 TG enhances survival rates in female *Drosophila* exposed to DSS

**FIGURE 1:**
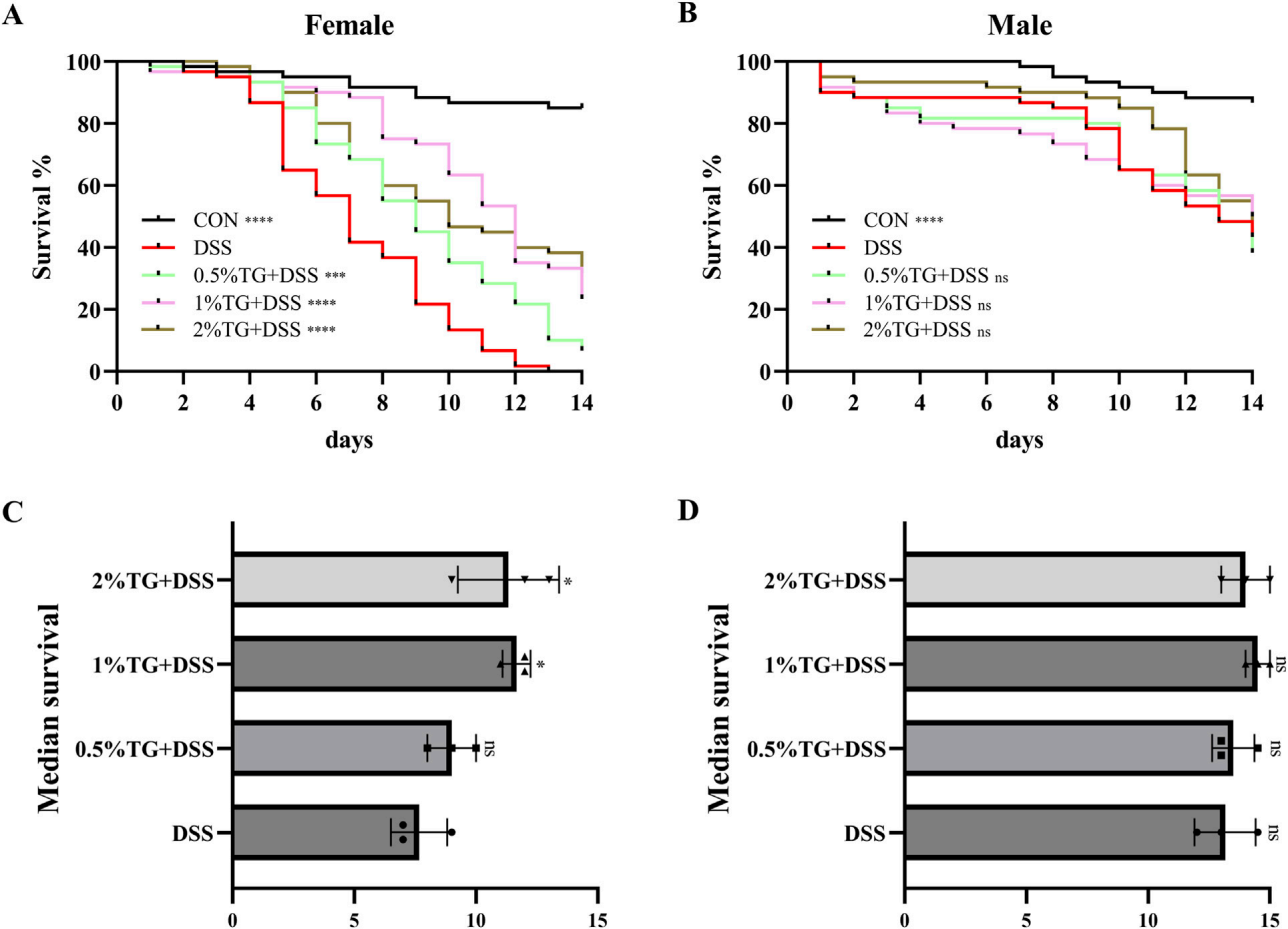
TG Increases Survival Rates in Female Fruit Flies with DSS-Induced Intestinal Injury. **(A)** Percentage survival rate of female fruit flies across groups. **(B)** Percentage survival rate of male fruit flies across groups. **(C)** The median lifespan in females. **(D)** The median lifespan in males. Significance levels indicate differences compared to the DSS-treated group: *P < 0.05, ***P < 0.001, ****P < 0.0001.

Survival analysis is commonly used to observe the potential effects of candidate compounds on stress recovery, lifespan, and related traits (Russi et al., 2020). Therefore, we assessed the effect of TG on the survival rate of *Drosophila* with DSS-induced UC (Figure 1). Female flies treated with 1% and 2% TG demonstrated significantly higher survival rates and median survival times compared to the DSS only group (*P* < 0.05; Figures 1A, C). However, under the same conditions, no significant differences in survival rate or median survival time were observed in male flies (Figures 1B, D). Collectively, these results suggest that TG exerts an anti-inflammatory effect in female *Drosophila* with DSS-intestinal injury. Therefore, subsequent investigations focused mainly on the protective effect of TG against DSS-induced UC in female flies.

### 3.2 TG alleviates DSS-induced intestinal damage

**FIGURE 2:**
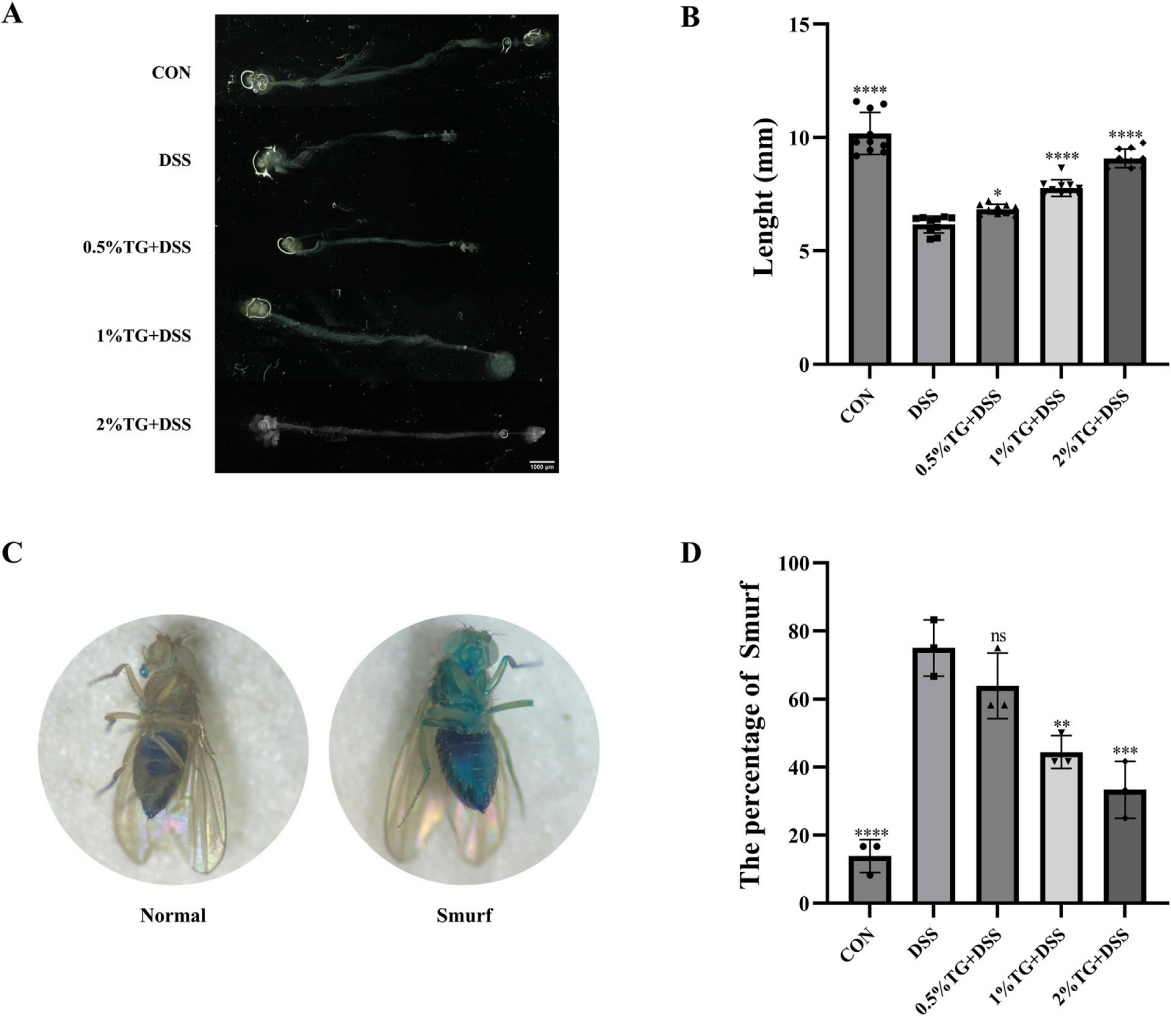
TG Mitigates Morphological and Barrier Integrity Damage in DSS-Induced Intestinal Injury. **(A)** Representative images of the *Drosophila* intestine **(B)** Quantitative analysis of intestinal length in **(A)** (n = 10). **(C)** Assessment of intestinal barrier function. Left: “Normal” flies; Right: “Smurfs” phenotype. **(D)** Percentage of Smurf phenotype flies significantly reduced in the groups fed with 1% and 2% TG compared to the DSS group (n = 36). Data are presented as mean ± SEM. *P < 0.05, **P < 0.01, ***P < 0.001, ****P < 0.0001, compared to the DSS group.

To investigate whether TG protects against DSS-induced morphological damage in the intestines of female *Drosophila*, we examined changes in intestinal length and permeability. Intestinal length was 39.40% longer in the control (CON) group (Figures 2A, B) than in the UC model group. In contrast, TG treatment significantly increased intestinal length in a dose-dependent manner (*P* < 0.05), with the group treated with 2% TG showing a 47.24% increase in intestinal length (Figures 2A, B). *Drosophila* intestinal epithelial cells are susceptible to DSS-induced damage, resulting in compromised barrier function and dye permeation throughout the fly body, quantified via the “Smurf” assay (Dambroise et al., 2016; Liu et al., 2016; Patel et al., 2019). Therefore, we performed “Smurf” assay to evaluate the protective effect of TG on intestinal integrity. Compared to that in the CON group, there was a marked increase in the percentage of “Smurf” flies in the UC model group, indicating increased intestinal permeability. However, treatment with 1% and 2% TG notably reduced the proportion of “Smurf” flies (*P* < 0.01), indicating that TG alleviated DSS-induced disruption of intestinal barrier (Figures 2C, D). Overall, these results suggest that TG protects against DSS-induced intestinal damage in *Drosophila*.

### 3.3 TG protects against DSS-induced apoptosis in intestinal epithelial cells

**FIGURE 3:**
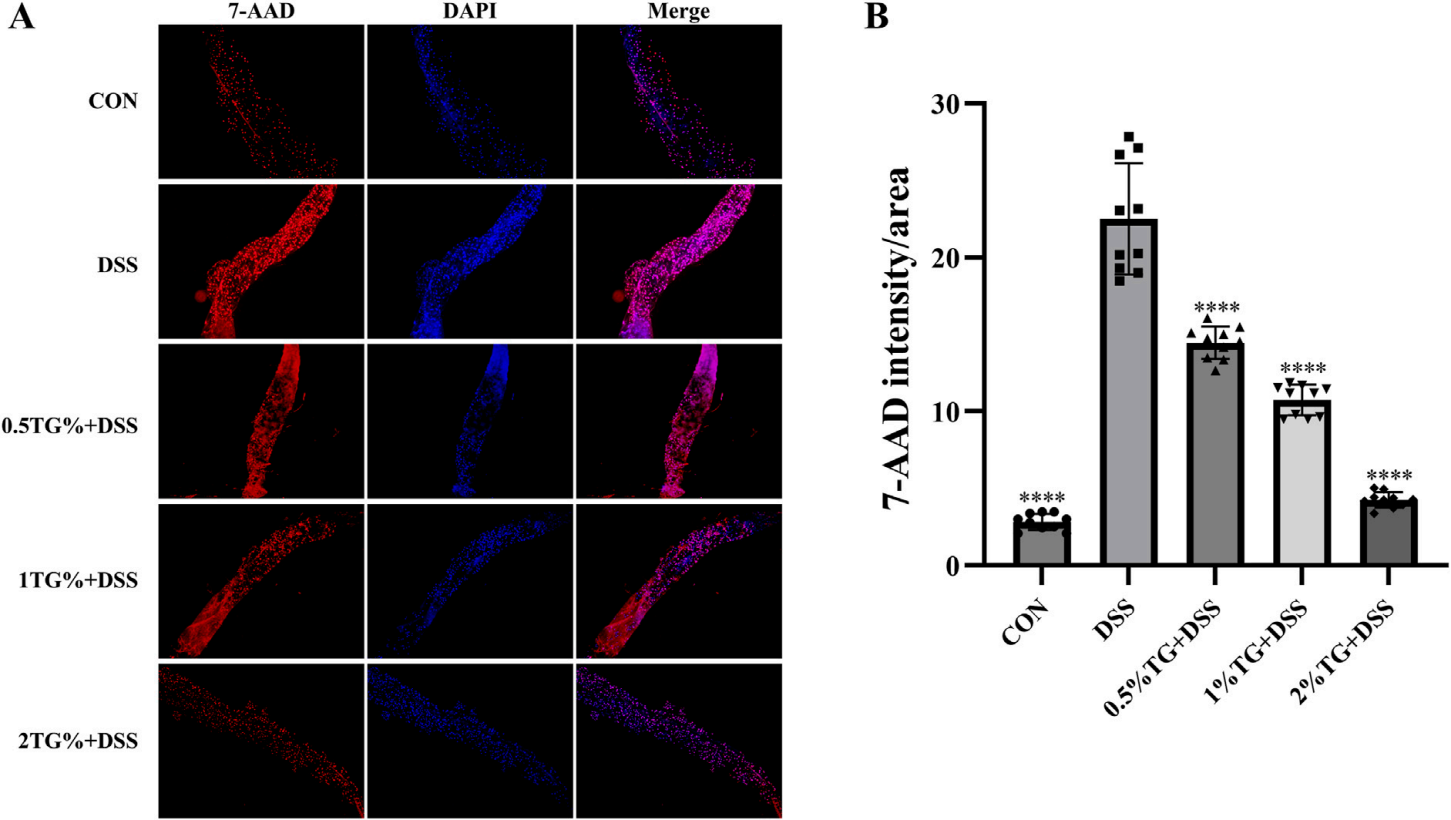
TG inhibits DSS-induced epithelial cell death in the intestine. **(A)** Representative images of fly intestines stained with 7-AAD and DAPI, indicating cellular death and nuclear morphology, respectively. **(B)** Quantitative analysis of fluorescence intensity in deceased cells across groups (n = 10). Data are presented as mean ± SEM. Significance compared to the DSS group is denoted as ****P < 0.0001.

In this study, 7-AAD staining was performed to assess DSS-induced apoptosis in intestinal epithelial cells. As indicated by fluorescence intensity, the number of apoptotic epithelial cells was significantly higher in the UC model group than in the CON group (*P* < 0.0001; Figure 3). However, TG treatment decrease epithelial cell apoptosis in a concentration-dependent manner, with no significant difference in cell apoptosis between the 2% TG and CON groups (Figures 3A, B). Overall, these findings suggest that high concentrations of TG alleviate DSS-induced apoptosis in intestinal epithelial cells.

### 3.4 TG protects against DSS-induced oxidative stress in the intestine

**FIGURE 4:**
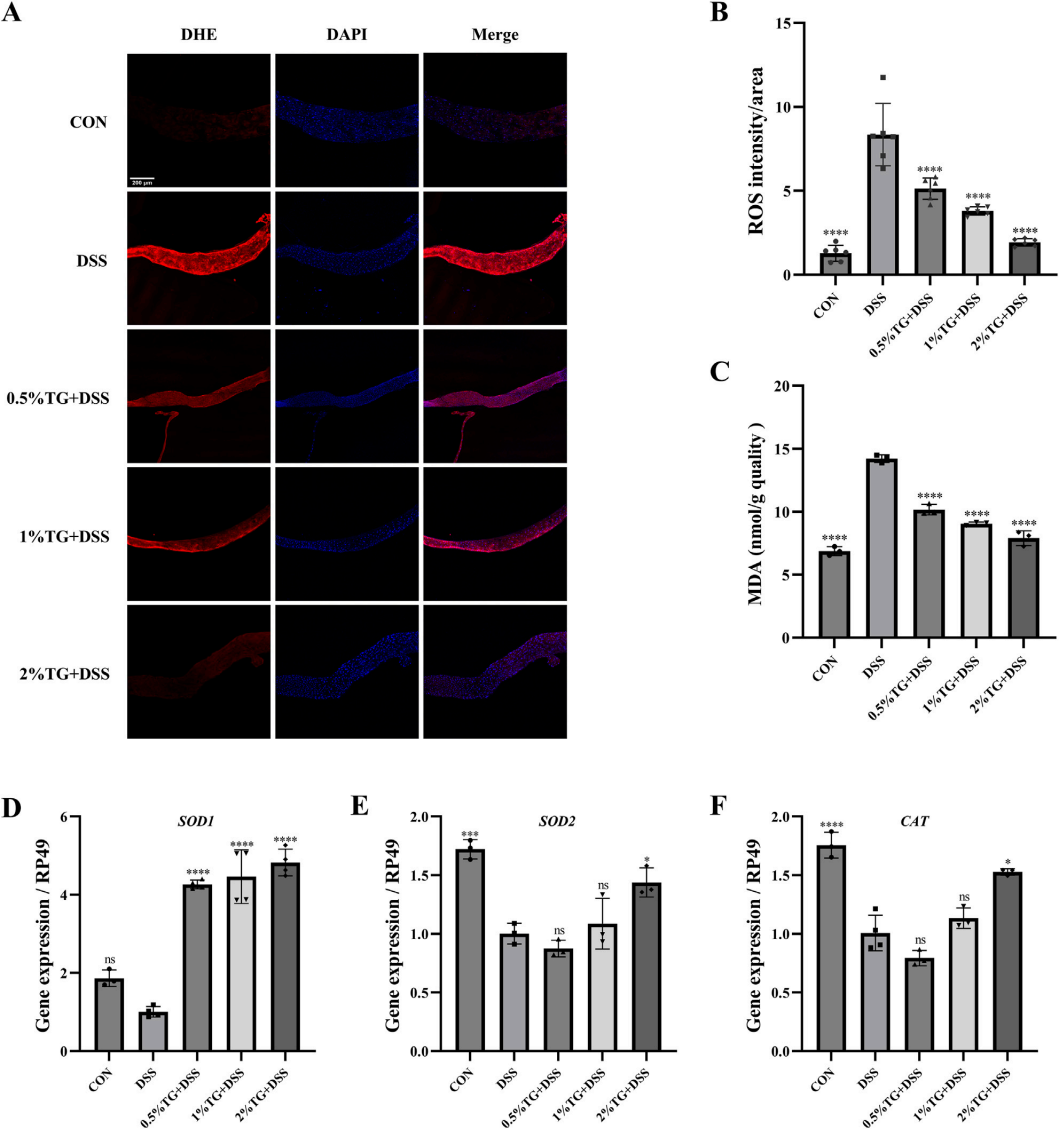
TG reduces oxidative stress in DSS-induced intestinal injury. **(A)** DHE and DAPI staining of *Drosophila* intestine to assess ROS accumulation. **(B)** Quantitative analysis of ROS levels via fluorescence intensity in the intestine (n = 6). **(C)** Measurement of MDA levels, an oxidative stress marker (n = 3). **(D–F)** mRNA expression levels of antioxidant genes *SOD1*, *SOD2*, and *CAT* in intestinal tissue (n = 3). Data are presented as mean ± SEM. Statistical significance compared to the DSS group: **P < 0.01, ***P < 0.001, ****P < 0.0001.

We used DHE probe to examine whether TG alleviates DSS-induced intestinal damage in adult female *Drosophila* by reducing oxidative stress. DHE staining intensity was higher in the UC model group than in the CON group, indicating increased oxidative stress. However, TG treatment significantly decreased DHE fluorescence intensity in a dose-dependent manner, with higher TG concentrations resulting in lower ROS levels (*P* < 0.0001; Figures 4A, B). Additionally, the level of MDA, a marker of lipid peroxidation damage (Wei et al., 2022), increased by 107.01% in the UC group compared with that in the CON group. However, treatment with all concentrations of TG significantly reduced MDA accumulation (*P* < 0.0001; Figure 4C). Overall, these findings suggest that TG ameliorates DSS-induced oxidative damage.

The Nrf2/Keap1 pathway serves as a protective mechanism against oxidative stress by promoting the expression of antioxidant enzymes, such as superoxide dismutase (SOD) and catalase (CAT) (Li et al., 2024). Therefore, we examined the mRNA levels of *SOD1*, *SOD2*, and *CAT* in the intestinal tissue of the flies. Notably, treatment with 2% TG significantly upregulated *SOD1*, *SOD2*, and *CAT* mRNA levels compared with those in DSS group (*P* < 0.05; Figures 4D–F). Overall, TG protects the intestine from DSS-induced oxidative damage by preserving redox homeostasis.

### 3.5 Network analysis prediction of the mechanism of TG in UC treatment

**FIGURE 5:**
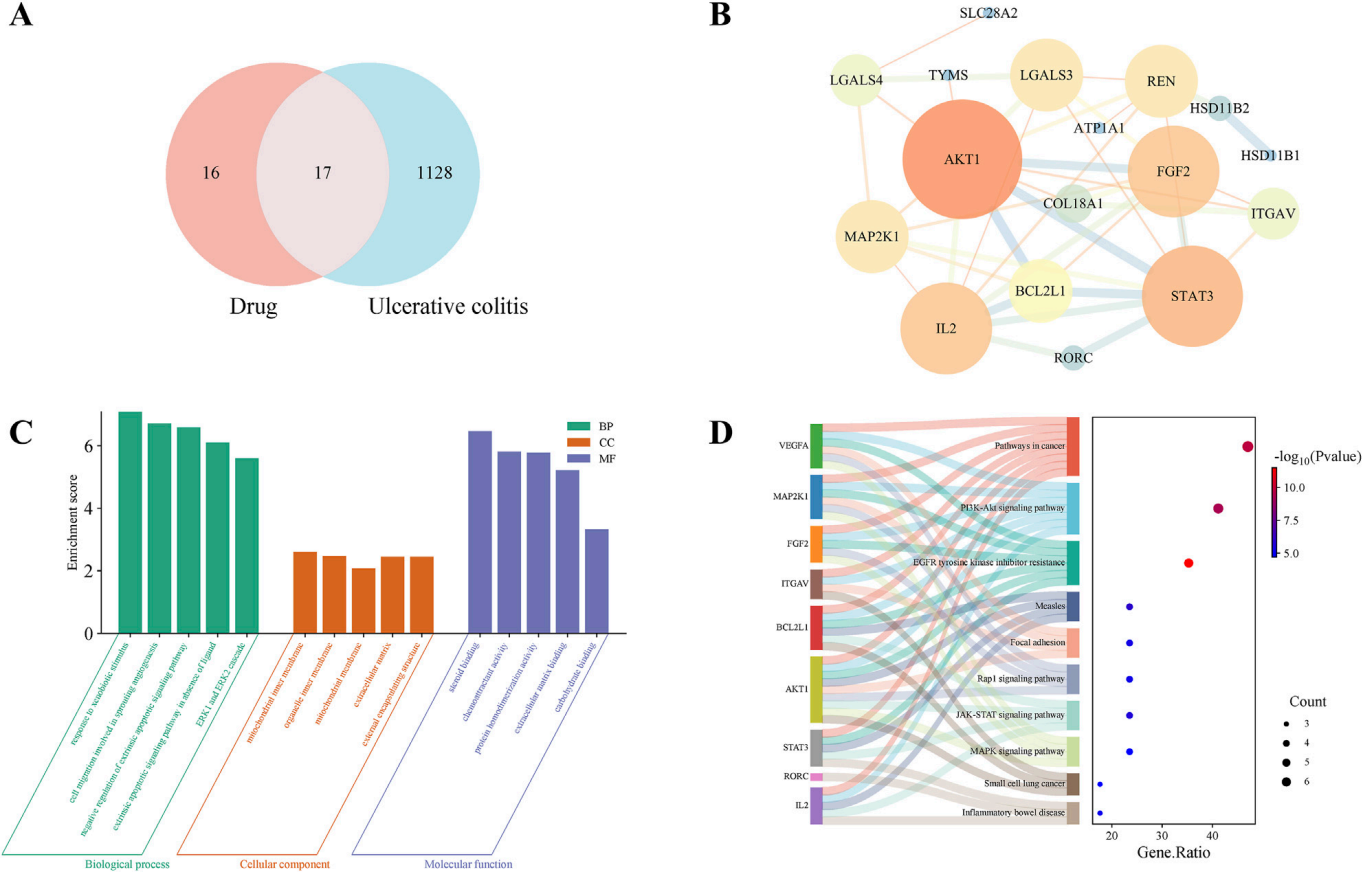
Network analysis-based prediction of potential mechanisms by which TG treats UC. **(A)** Venn diagram illustrating overlapping targets between TG and UC. **(B)** Protein-protein interaction (PPI) network of TG-UC targets. **(C)** GO enrichment analysis of biological processes associated with TG-UC targets. **(D)** KEGG signaling pathways enriched in TG-UC target interactions.

Network analysis was performed to predict potential important targets and mechanisms. To date, over 200 distinct ginsenosides have been reported, including Rb1, Rb2, Rc, Rd, Re, Rf, and Rg1, which constitute over 90% of the TG in raw ginseng roots (Jakaria et al., 2018; Mohanan et al., 2018). Notably, we identified the targets of these seven primary ginsenosides using the Swiss target prediction databases and screened 33 drug targets (Figure 5A). Additionally, we screened for UC-related disease targets using the GeneCards, OMIM, TTD, DrugBank, and PharmGBK databases. After unification and deduplication using UniProt, we identified 1,145 disease-associated targets in UC. Importantly, we identified 17 potential overlapping drug-disease targets based on the intersection of the drug and disease targets in a Venn diagram (Figure 5A). To further analyze the core targets of TG in UC treatment, we imported the data into the STRING database and used Cytoscape software to construct and analyze the PPI network. Notably, We identified 39 UC-related targets, and based on network topology analysis, *AKT1* and *STAT3* were predicted to be core targets (Figure 5B). Additionally, we performed KEGG and GO enrichment analyses of the consensus targets using Metascape (Figures 5C, D). The results were arranged based on the p-value. GO analysis showed that the targets were primarily enriched in the following biological processes: responses to xenobiotic stimuli, negative regulation of the extrinsic apoptotic signaling pathway, and regulation of the ERK1/ERK2 cascade. Additionally, the targets were enriched in the cytoplasm, organelles, and mitochondria in the “cellular component” category, and in molecular functions associated with steroid binding, chemoattractant activity, and protein homodimerization activity. KEGG pathway analysis indicated that the targets were mainly enriched in pathways related to EGFR tyrosine kinase inhibitor resistance and the MAPK, JAK/STAT, and PI3K/Akt pathways. Among these, the JAK/STAT and PI3K/Akt pathways are particularly important in UC research, with *AKT1* and *STAT3* being identified as key targets within the PPI network. Based on these results and previous findings (Li et al., 2024; Surbek et al., 2021), it can be concluded that TG exerts its effects in UC possibly via regulation of the JAK/STAT and PI3K/Akt signaling pathways.

### 3.6 TG mitigates DSS-induced intestinal injury by regulating the expression of related genes in the PI3K/Akt and JAK/STAT pathways

**FIGURE 6:**
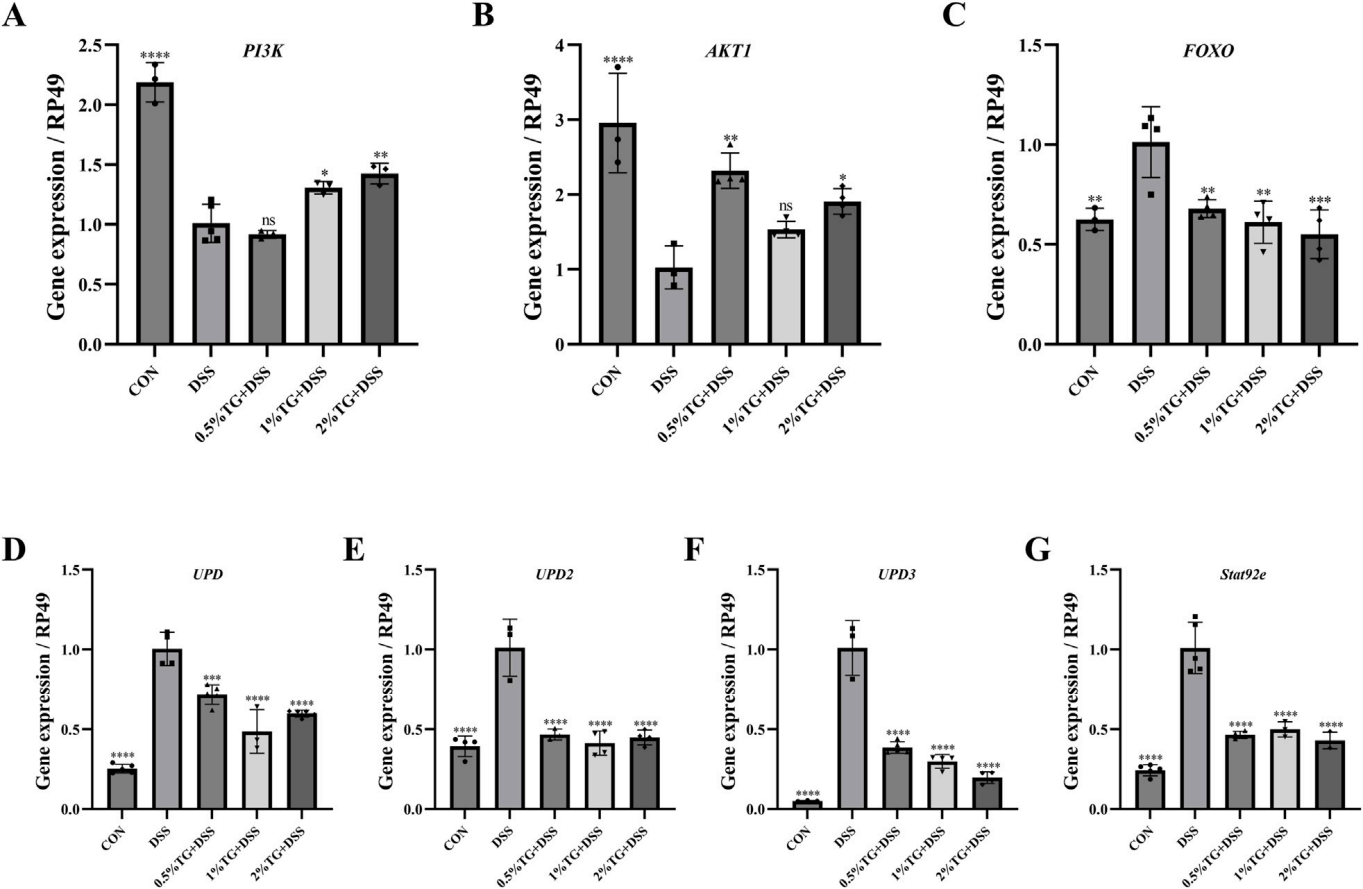
TG alleviates DSS-induced intestinal injury in *Drosophila* through modulation of the PI3K/Akt and JAK/STAT signaling pathways. **(A–C)** Expression levels of cytokines and genes related to the PI3K/Akt signaling pathway in the intestines of adult female *Drosophila* (n = 3). **(D–G)** Expression of genes associated with the JAK/STAT signaling pathway in the intestines of adult female *Drosophila* (n = 3). Data are presented as mean ± SEM. Significance compared to the DSS group: *P < 0.05, **P < 0.01, ***P < 0.001, ****P < 0.0001.

Considering that network analysis identified the PI3K/Akt and JAK/STAT signaling pathways as the potential mechanisms underlying the therapeutic effects of TG in UC, we examined the expression of genes related to the PI3K/Akt pathway, including *PI3K*, *AKT1*, and *FOXO*. Compared with those in the DSS group, TG treatment upregulated *PI3K* and *AKT1* mRNA expression and significantly downregulated the pro-apoptotic gene *FOXO* (*P* < 0.01; Figures 6A–C). Collectively, these results suggest that TG activates the PI3K/Akt pathway, thereby inhibiting *FOXO* expression and reducing intestinal epithelial cell apoptosis and inflammation. To confirm that TG alleviated DSS-induced intestinal damage *via* the JAK/STAT pathway, we examined the expression of *UPD*, *UPD2*, *UPD3*, and *Socs92e*. Although DSS stimulation markedly increased the mRNA levels of *UPD*, *UPD2*, *UPD3*, and *Socs92e* in the intestine, TG treatments significantly decreased the expression of these genes (*P* < 0.001; Figures 6D–G). Overall, these findings indicate that TG may help maintain intestinal homeostasis by activating the PI3K/Akt and inhibiting the JAK/STAT signaling pathway.

## 4 Discussion

UC is a chronic, recurrent inflammatory bowel disease with poorly understood pathogenesis that often presents treatment challenges (Le Berre et al., 2023). Recently, there has been a growing interest in the use of natural, nontoxic plant metabolites to prevent or alleviate UC symptoms. TG, the primary bioactive metabolite in *Panax ginseng*, has shown promising anti-inflammatory, antioxidant, and immunomodulatory properties in various disease models (Ratan et al., 2021). However, studies on the specific mechanisms by which TG modulate intestinal injury, particularly UC, are limited. Therefore, we investigated the therapeutic effect and potential molecular mechanisms of TG in UC using a *Drosophila* model of DSS-induced intestinal inflammation and network analysis.

Survival analysis is widely used to assess toxicity and determine the optimal concentrations of potential therapeutic candidates in experimental models (Xiu et al., 2022). In this study, TG treatment significantly improved the survival rate of female flies with DSS-induced UC, but did not increase the survival rate of male flies. Although all DSS-treated female flies succumbed, the mortality rate among the male flies was only 51.7%, Sex-based differences in inflammatory and immune responses in *Drosophila* may contribute to these results (Wagnerova et al., 2017). Overall, these findings suggest that male flies possess stronger resistance to DSS-induced toxicity, resulting in milder intestinal injury and, thus, a diminished therapeutic benefit from TG. Consequently, future research will prioritize investigating the protective mechanisms of TG in mitigating intestinal damage in female *Drosophila*. Maintaining intestinal homeostasis depends on the integrity of the physical barrier of the intestinal tissue, and this integrity is compromised under stress (He et al., 2022). Our study showed that TG treatment significantly increased the intestinal length of flies with DSS-induced UC and markedly reduced the proportion of “Smurf” stain, suggesting that TG alleviates DSS-induced intestinal damage. Toxic chemicals, such as DSS, reportedly harm intestinal cells and disrupt epithelial homeostasis (He et al., 2022; Zhang et al., 2022). Our findings support this by showing that TG supplementation is effective in reducing intestinal epithelial cell death. In addition, the Nrf2/Keap1 pathway is a key antioxidant mechanism, and activation of this pathway increases the expression of antioxidant enzyme genes, reduces ROS accumulation, and attenuates DSS-induced intestinal damage (Yang et al., 2022). This is supported by our experimental results, which showed that TG supplementation significantly upregulated the expression levels of *SOD1*, *SOD2* and *CAT* genes in the intestines of DSS-treated flies. ROS play an important role in maintaining intestinal homeostasis. Excessive ROS can trigger oxidative stress and lead to intestinal dysfunction (Chen et al., 2021). According to our study, TG successfully reduced the accumulation of ROS in the midgut of DSS-treated flies. An important indicator of oxidative stress is MDA, which is a side effect of lipid peroxidation induced by cell membrane damage (Yang et al., 2024). Our study showed that TG supplementation reduced DSS-induced MDA levels in the fly intestine. Thus, TG protects the intestine by attenuating oxidative stress.

To further elucidate the mechanisms by which TG improves UC, we first employed network analysis to identify the PI3K/AKT and JAK/STAT signaling pathways as potential key pathways for TG to treat UC. Subsequently, experimental validation was performed to confirm the involvement of these pathways. The PI3K/AKT signaling pathway is fundamental for the regulation of cell proliferation, metabolism, growth, differentiation, and apoptosis (Cao et al., 2022). Consistent with previous findings, TG protected against DSS-induced intestinal damage by upregulating *PI3K* and *Akt1* mRNA expression and downregulating *FOXO* mRNA expression in the intestine. Moreover, the JAK/STAT signaling pathway is a critical regulatory pathway for suppressing ISC hyperproliferation under inflammatory conditions. For example, safranal extract alleviated SDS-induced ISC proliferation by inhibiting JAK/STAT signaling (Lei et al., 2022). Similarly, TG suppressed DSS-induced ISC hyperproliferation by downregulating *UPD, UPD2, UPD3*, and *Socs92e* mRNA expression in this study.

However, the *Drosophila* model is a preliminary experimental system that differs from mammals in terms of pathological conditions, drug absorption, and metabolism. Therefore, further research utilizing more advanced models is necessary to compare the pharmacokinetics of TG in both healthy and UC-affected animals. The findings presented here provide a foundational basis for future investigations. In addition, although network analysis offers valuable preliminary insights into the potential pharmacological targets of TG, it involves complex mixtures, which limits the ability to assess the therapeutic effects of individual ginseng saponins on UC from a pharmacological perspective. Moreover, regarding mechanistic exploration, the absence of protein-level validation precludes definitive establishment of causal relationships for the proposed PI3K/Akt, and JAK/STAT pathways. To address these challenges, future studies should incorporate more rigorous experimental validation to ensure a more reliable evaluation of TG’s therapeutic potential.

## 5 Conclusion

In this study, we explored the therapeutic effects and molecular mechanisms of TG in UC using network analysis and a *Drosophila* model of DSS-induced intestinal injury. TG treatment significantly improved survival, reduced intestinal damage, and alleviated oxidative stress. Mechanistically, TG protects against intestinal injury by modulating the PI3K/Akt signaling pathway and inhibiting the JAK/STAT signaling pathway. Network analysis highlights TG’s potential for multi-target regulation of UC. These findings provide a foundation for further clinical exploration of TG as a potential therapeutic for UC.

## Data Availability

The original contributions presented in the study are included in the article, further inquiries can be directed to the corresponding author.

## Abbreviations

TG, Total Ginsenosides; UC, Ulcerative Colitis; IBD, Inflammatory Bowel Disease; DSS, Dextran Sulfate Sodium Salt; DAPI, 4,6-Diamidino-2-phenylindole; DHE, Dihydroethidium; 7-AAD, 7-Aminoactinomycin D; PBS, Phosphate buffered saline; ROS, Reactive Oxygen Species; MDA, Malondialdehyde; PPI, Protein-Protein Interaction; GO, Gene Ontology; KEGG, Kyoto Encyclopedia of Genes and Genomes; SOD, Dismutase; CAT, Catalase; ISC, Intestinal Stem Cell.
